# Acupuncture and Moxibustion for Cancer-Related Fatigue: An Overview of Systematic Reviews and Meta-Analysis

**DOI:** 10.3390/cancers14102347

**Published:** 2022-05-10

**Authors:** Tae-Young Choi, Lin Ang, Ji Hee Jun, Terje Alraek, Myeong Soo Lee

**Affiliations:** 1 KM Science Research Division, Korea Institute of Oriental Medicine, Daejeon 34054, Korea; superoung@kiom.re.kr (T.-Y.C.); anglin2808@gmail.com (L.A.); zhixi04@kiom.re.kr (J.H.J.); 2 School of Health Sciences, Kristiania University College, 0107 Oslo, Norway; terje.alrak@uit.no; 3 The National Research Center in Complementary and Alternative Medicine (NAFKAM), Department of Community Medicine, Faculty of Health Science, UiT, The Arctic University of Norway, 9037 Tromsø, Norway

**Keywords:** complementary medicine, acupuncture, cancer, fatigue, systematic review

## Abstract

**Simple Summary:**

Acupuncture, per se, is not used for treating cancer. However, acupuncture is used for treating several cancer-related symptoms, such as, for example, pain, antiestrogen-induced hot flashes, as well as cancer-related fatigue (CRF). There are several studies that assess the evidence of acupuncture for palliative cancer treatment; but there are none for CRF. The aim of this overview, therefore, was to comprehensively summarize and critically evaluate the current evidence of the efficacy of AT in the management of CRF.

**Abstract:**

Although acupuncture (AT) is used in the treatment of CRF, the evidence from different systematic reviews (SRs) of AT has not yet been comprehensively evaluated. Moxibustion, which is a treatment method that is well established within Traditional East Asian Medicine, applies the heat of burning herbs towards or onto special points on the skin. Commonly, the herb Artemisia vulgaris, is used. It has been used for palliative cancer care, as well as for CRF. The aim of this overview was to evaluate the efficacy of AT and moxibustion in the management of CRF. Eleven databases were searched through for studies that were published from their dates of inception to February 2022. The study selection, the data extraction, and the assessment were performed independently by two researchers. The methodological and report quality were assessed by using the Assessment of Multiple Systematic Reviews-2 (AMSTAR-2) tool. The evidence quality was evaluated by using the Grading of Recommendations Assessment, Development and Evaluation (GRADE) system. Fifteen SRs on AT (n = 10) and moxibustion (n = 5) treatments for CRF were included, and they include 169 randomized controlled trials and 14,392 participants. All of the SRs that were evaluated by the AMASTAR-2 had more than one deficiency, and so all of the SRs were rated as either low or critically low. For the GRADE, 18 outcomes were rated as very-low-quality evidence, 13 as low-quality evidence, 3 as moderate-quality evidence, and 0 as high-quality evidence. Most of the SRs reached the potential benefits of AT for CRF. No serious adverse effects were identified. In conclusion, the evidence suggests that, despite the advantages of AT in terms of the improvement in and the safety of the treatment of CRF, the methodological quality of most of these studies is low, which limits our ability to draw definitive meanings. Further research of high quality is needed in order to confirm these findings.

## 1. Introduction

One of the most frequent side effects of chemotherapy and radiation therapy is cancer-related fatigue (CRF). The exact reason for CRF has yet to be determined. It is associated to the illness process, as well as to treatments such as surgery, chemotherapy, and radiation therapy. Fatigue could be a side effect of any chemotherapy drug, and it is commonly caused by medications such as vincristine, vinblastine, and cisplatin [1]. As it includes physical, mental, and emotional aspects, CRF is a multidimensional symptom, and it can have a significant impact on the patient’s life. Even after cancer treatment, some people are unable to return to or engage in their usual daily activities. Both the patient and family members often experience tremendous stress and anxiety that are due to CRF [2].

The symptoms of CRF are underreported by patients, and underestimated and undertreated by clinicians, despite the prevalence and negative impact of CRF. Cancer patients usually start to exhibit fatigue symptoms during or after treatment, and these may last for anywhere between 2 weeks and 5 years [3,4]. In cancer treatment, quality-of-life (QoL) factors are the second most important treatment goal after the survival rate. CRF can be distinguished from general fatigue in that it is not relieved by rest, and it is not primarily induced by physical activity [5]. Overall, 50–90% of cancer patients who undergo treatment experience CRF [6,7]. This results in a significant deterioration in the QOL of cancer patients, as well as of their families, with crippling social and economic consequences [8].

There is no effective standard medical treatment for CRF and, hence, cancer patients are also using alternative and complementary therapies for their CRF [9]. Currently, nonpharmacological interventions are the main approaches to managing CRF. Among them, there is emerging evidence that different forms of exercise are an option for treating CRF [10]. Furthermore, the National Comprehensive Cancer Network (NCCN) guidelines include meditation, muscle relaxation, yoga, Tai Chi, cognitive behavioral therapy, and acupuncture to relieve symptoms of CRF [5,11,12]. However, these therapies often require the subjective cooperation of patients to increase the therapeutic compliance. Therapeutic compliance not only includes patient compliance with medication, but also with diet, exercise, or lifestyle changes.

Recently, complementary approaches, such as acupuncture and moxibustion, as an alternate strategy for CRF symptom management has been an area of increased scrutiny. Acupuncture and moxibustion are two complementary therapeutic methods: the former uses a needle to stimulate an acupoint, while the latter uses heat that is generated by moxa burning. Acupuncture is recommended by the NCCN guidelines, and especially for patients who have finished anticancer treatment and are defined as cancer survivors. It has been tested for safety and efficiency by a number of randomized controlled trials (RCTs) [10,11,12,13]. Moxibustion, on the other hand, has been used for palliative cancer care, as well as for CRF. Acupuncture is considered to be a safe treatment in the hands of qualified health personnel [13,14,15]. However, cancer patients are a vulnerable group (i.e., they have lower immune responses) and, therefore, needling the arm is avoided in patients who have undergone axillary dissection, and the same applies for any lymphedematous limbs. Hence, special precautions are needed with regard to safety in order to prevent adverse effects [16]. In line with this, the present study will address adverse events. In addition, systematic reviews (SRs) for the evaluation of the effects of AT are also increasing [17,18,19]. However, there have been previous studies on the effect of acupuncture on CRF that have yielded controversial results. There is a detailed overview of the systematic reviews on acupuncture for cancer palliative treatment [20], but there are none for CRF. The aim of this overview, therefore, was to comprehensively summarize and critically evaluate the current evidence from SRs to determine the efficacy of AT in the management of CRF. 

## 2. Methods

### 2.1. Study Registration

This protocol was registered on the Research Registry, with the registration number: reviewregistry1252. 

### 2.2. Database and Search 

We searched 12 databases, including the English, Chinese, and Korean databases of Pubmed, EMBASE, the Cochrane Database of Systematic Reviews, Chinese National Knowledge Infrastructure (CNKI), Wanfang Databases, Korea Med, the Oriental Medicine Advanced Search Integrated System (OASIS), DBpia, the Korean Medical Database (KM base), the Research Information Service System (RISS), and the Korean Studies Information Services System (KISS), from inception to February 2021. There were no restrictions on the language or the publication status. 

The search terms that were used are as follows: (“acupuncture” OR “electro-acupuncture” OR “electroacupuncture” OR “auricular acupuncture” OR “moxibustion” OR “acupressure” OR OR “acupuncture points”) AND (“cancer related-fatigue” OR “CRF” OR “cancer fatigue”) AND (“systematic review” OR “meta-analysis” OR “meta analysis”). Additional studies were identified through the reference lists in the included SRs. 

### 2.3. Eligibility Criteria 

#### 2.3.1. Types of Studies

Those SRs and meta-analyses of RCTs that used acupuncture and moxibustion for CRF were eligible. There was no language restriction.

#### 2.3.2. Types of Participants

Patients diagnosed with CRF, regardless of gender, age, or the cause of the CRF, were eligible. There were no restrictions on the cancer stage or the pathology subtypes.

#### 2.3.3. Types of Interventions and Comparators 

Acupuncture, including electro-acupuncture, auricular acupuncture, warm-acupuncture, dry needling, and other active treatments, and moxibustion studies that were used to treat CRF, were included. Studies that compared a combination of acupuncture, moxibustion, and another active treatment versus other active treatments alone were also eligible. The comparator groups that were eligible were standard medication, sham acupuncture, standard care, or no intervention/wait list.

#### 2.3.4. Types of Outcome Measures

The clinical efficiency (total effective rate or cure rate; clinical symptom integral), and rating scales that describe the fatigue score, were accepted as the primary results. The QOL improvement rate was a secondary outcome.

#### 2.3.5. Exclusion Criteria 

The exclusion criteria were: (1) SRs of non-RCT study designs; (2) SRs that included laser acupuncture, acupoint injection, or transcutaneous electrical nerve stimulation (TENS); (3) SRs that compare the efficacy of different acupuncture modalities; and (4) SRs with meta-analyses that did not synthesize original data.

### 2.4. Studies Selection 

Two authors (T.-Y.C. and L.A.) worked independently on the selection process. First, according to the inclusion and exclusion criteria, all of the identified titles and abstracts for potentially relevant studies were evaluated. Next, the full texts of the eligible inclusion articles were retrieved for further examination. Any conflicts were settled by discussion with a third author (M.S.L.) at each stage of the process.

### 2.5. Data Extraction

Two authors (T.-Y.C. and L.A.) extracted the data independently, and any discrepancies were resolved by discussion with a third author (M.S.L.). The findings of the literature search and the data extraction were comprehensively summarized. The author; publication year; search date; number of searched databases; number of primary studies, including total sample size; type of intervention and comparator; outcome measures; quality assessment tool; overall risk of bias; effect estimates (meta-analysis) for main outcomes; conclusions that were quoted from the original article; and adverse events were all extracted from the included SRs. 

### 2.6. Quality Assessment

Two authors (T.-Y.C. and L.A.) used the Assessment of Multiple Systematic Reviews-2 (AMSTAR-2) tool [21] to evaluate the quality of reporting in each systematic review that was included. Differences in study assessments were resolved through discussion and through consultation with a third author (M.S.L.).

With evaluations of “yes”, “partial yes”, or “no”, the AMSTAR-2 is an approved 16-item tool that is used for objectively assessing SRs. The confidence in the SR results were rated overall into four categories: “High” (no flaws or one noncritical flaw); “Moderate” (more than one noncritical flaw); “Low” (one critical flaw, with or without noncritical flaws); and “Critically low” (more than one critical flaw, with or without noncritical flaws). Since the AMSTAR-2 does not generate an overall “score”, and since several critical weaknesses of an SR may be disguised, a process of considered judgment was used to interpret the AMSTAR-2 results, until a consensus was reached on the overall methodological quality of the included SRs. 

### 2.7. Certainty of Evidence (CoE)

The CoE for each outcome was assessed by using the Grading of Recommendations Assessment, Development, and Evaluation (GRADE: GRADEpro Guideline Development Tool [Software] McMaster University and Evidence Prime, Inc. 2015. Ontario, Canada) approach. The GRADE method divides the CoE into four categories: high, moderate, low, and very low. On the basis of RCTs, the evidence was initially given a high rating, and it was gradually downgraded, or even upgraded, because of the following: (1) risk of bias; (2) evidence indirectness; (3) heterogenous results or inconsistency; (4) result imprecision; and (5) publication bias. Two reviewers (T.-Y.C. and L.A.) independently analyzed the evidence and their relevant outcomes. Any upgraded or downgraded aspects that affected the quality of evidence were explained in detail in order to provide a transparent and reliable result. Any discrepancies were worked out with the help of a third author (M.S.L.) through discussion. 

### 2.8. Data Analysis

As the data from the RCTs were overlapped with SRs, a quantitative analysis of the SRs was not performed. A qualitative analysis of the same investigations, on the other hand, was presented. All findings were descriptively summarized and reported as a narrative analysis. The effects of the interventions were also computed. The methodological quality of the SRs, as well as the quality of the evidence for the outcomes, were also tabulated. We analyzed the frequency of acupuncture points, the CRF scales, and the interventions that were used in the primary studies of the included SRs.

## 3. Results

### 3.1. Study Identification

**Figure 1:**
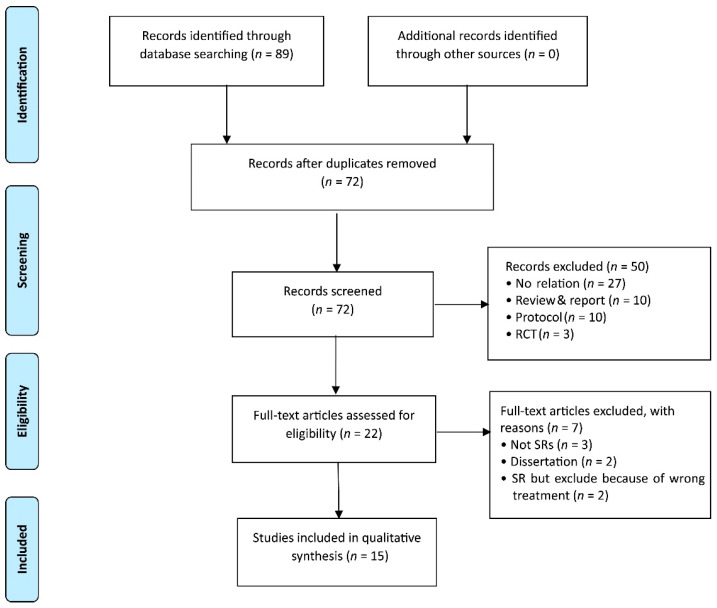
Flow chart of study selection. RCT: randomized control trial. SR: systematic review.

A total of 89 eligible studies were identified, and a total of 17 duplicated studies were deleted. A total of 50 studies were excluded on the basis of the title or the abstract being irrelevant to the topic. After screening through the full-text reviews, 15 studies [17,18,19,22,23,24,25,26,27,28,29,30,31,32,33] were finally selected for analysis (Figure 1). The excluded articles and the reasons for the exclusions in “full-text assessed for eligibility” are shown in the Table S1.

### 3.2. Characteristics of Included Studies

All SRs were published between the years of 2013 and 2021, and there were 11 SRs from China [17,18,19,24,25,27,28,29,30,31,32], 2 SRs from Korea [26,33], and 2 SRs from Australia [22,23] (Table 1). 

A total of ten studies were published in English [17,18,19,22,23,25,26,27,28,33], and five were published in Chinese [24,29,30,31,32]. All of the SRs/meta-analyses that were used contained RCTs. Various types of cancer patients were involved. Fifteen of the SRs represent from 4 to 28 RCTs, and from 374 to 2249 participants. The interventions in the therapy group were mainly acupuncture (n = 10) [17,18,19,22,23,24,25,26,27,28] and moxibustion (n = 5) [29,30,31,32,33], and the control group was made up mainly of conventional medicine (usual care) and sham AT. The usual care described in the primary studies included the patients’ cancer education, psychological counselling, regular exercise, diet care, and improvement in sleep routine. Fourteen SRs used the Cochrane Risk of Bias (RoB) tool for the methodological quality assessment. Only one SR-quality-assessment tool was not mentioned [25]. A meta-analysis was performed in 13 SRs/meta-analyses. Only 2 SRs were not meta-analysed [25,26]. Most of the studies supported the idea that acupuncture and moxibustion could improve CRF. The top acupoints that were selected in the primary studies were ST36, followed by CV6, CV4, SP6, CV12, CV8, and LI4 (Table 2). The outcome indicators were related to fatigue and to QoL. The fatigue scoring that was used in most of the primary studies that were included in the SRs were the Brief Fatigue Inventory (BFI) and the Piper Fatigue Score (PFS), which were followed by the Functional Assessment of Cancer Treatment (FACT), the Multidimensional Fatigue Inventory (MFI), etc.

### 3.3. Assessment of Quality 

**Figure 2:**
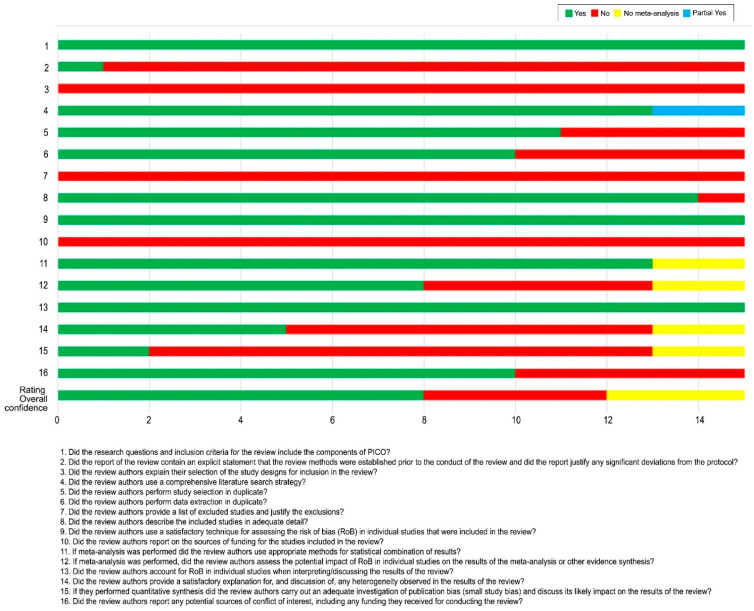
Methodological and reporting quality (evaluation results of each AMSTR-2). AMSTAR-2: Assessment of Multiple Systematic Reviews-2.

The results of the AMSTAR-2 assessment are presented in Figure 2. The assessment was not designed to generate an overall “score” that would disguise the critical weaknesses in specific domains. 

Three SRs were of moderate quality, four were of low quality, and eight were of critically low quality, according to the AMSTAR-2 (Figure 2, Table S2). All of the SRs used adequate tools to assess the risk of bias (Item 9); however, only one study establishes a prior study protocol (Item 2). None of the SRs explain the reasons for the study-type selection or provide a complete list of the excluded studies with reasons (Items 3 and 7). None of the studies report the sources of funding for the studies that were included in the SRs (Item 10). Only a few SRs assessed the publication bias with a funnel plot (Item 15), whereas five studies do not report any probable sources of conflicts of interest (Item 16).

### 3.4. Certainty of Evidence (CoE) 

Thirty-four outcomes were examined, and none of them had high-quality evidence. According to the GRADE evaluation, the quality of the evidence was moderate in 3 outcomes (8.8%), low in 13 outcomes (38.2%), and extremely low in 18 outcomes (53.0%), as is summarized in Table 3. 

### 3.5. Effectiveness of Acupuncture for CRF

A total of 15 SRs summarized the evidence on the effectiveness of acupuncture and related therapies (acupressure, moxibustion) in the treatment of CRF, and 13 SRs conducted meta-analyses (Table 2). The outcomes from the included SRs are summarized and presented in Table 3. 

The evidence in 13 SRs (26 outcomes) suggests that the fatigue of AT for CRF in various types of cancer patients was the superior control group (UC or Sham AT); however, it is uncertain whether the outcomes were assessed with validated scales. Acupuncture had better fatigue than the control group at follow-up [24]. Moreover, the QoL was reported in six SRs [24,27,28,29,30,32], and the meta-analysis showed that there was a statistical significance between the AT or moxibustion groups and the control group. One SR reported the TCM symptom score [30], and, by comparing the effects of the AT versus the control group results, showed that the AT treatment had a greater effect than the control group. Readers should note that all four of the trials that were included in this meta-analysis had poor reporting quality, and so their RoBs were unclear.

### 3.6. Adverse Events (AEs)

Of all of the 15 SRs, 10 SRs mentioned/registered the adverse events of acupuncture in the treatment of CRF [17,18,19,22,27,28,29,30,31,33], which included spot bleeding, bruising, discomfort, and nausea; burns with a mild blister; and dizziness. No serious events were directly correlated with AT and moxibustion. Nevertheless, because of the small samples that were included in the review, and because of certain missing data, we have no means of claiming the safety of acupuncture and moxibustion.

## 4. Discussion

This overview identified and evaluated all of the available evidence for the use of AT in the treatment of CRF. The findings of this overview were summarized according to the type of outcomes, and the GRADE was used to evaluate the quality of the identified evidence. A total of 15 SRs were identified, which comprise evidence from 169 primary RCTs, with a total of 14,392 patients. According to the clinical evidence that is included in this overview, AT-related therapies are likely to be beneficial in the treatment of CRF. Most of the SRs were rated as either low or critically low in the AMSTAR-2 evaluation, as they had more than one significant flaw. A total of eight out of fifteen SRs were assessed as being of critically low quality, four were rated low quality, and three were rated moderate quality, according to our AMSTAR-2 assessment. The lack of SR protocols or registrations, the omitted list of excluded research with reasons, the reporting of funding sources for the primary studies, and conflicts of interest were the key issues. In the included SRs/MAs, there was a lot of clinical heterogeneity in terms of the interventions and outcomes (e.g., treatment course, efficacy criteria, etc.). 

As the AMSTAR-2 assessment was performed by only relying on information that was accessible in the publications of SRs/MAs, it is also highly dependent on the quality of the primary studies that were included in the SRs/MAs. High-quality SRs/MAs cannot be conducted without high-quality RCTs. Although most RCTs support the use of acupuncture and related therapies in the management of CRF, the reporting quality of these studies is generally poor, which makes it difficult to estimate the risk of bias in these RCTs. Consolidated Standards of Reporting Trials (CONSORT) suggestions for reporting should be followed in future confirmatory trials. The evidence quality was also poor in the GRADE assessment. We also identified that the major reason for the downgrading of the evidence in the GRADE assessment was inconsistencies in the studies, as most RCTs failed to describe the blinding method, randomization, or evidence of considerable interstudy heterogeneity [34]. Therefore, this highly limits our ability to make unambiguous conclusions about the effectiveness of the CRF.

Because of the variation in the outcome measures, the interpretability of the results is limited. The BFI and PFS were used for measuring CRF in most of the primary studies, while it was presented as a response rate in others. Future trials should choose the most relevant endpoint as the primary outcome, and they should measure it by using a proven manner in order to assure the clinical evidence’s utility in the future.

The most frequently used acupuncture point was ST36. Acupoint ST36 has the ability to tonify qi and blood, and it is mainly used to improve spleen-deficiency syndrome [35]. ST36, in particular, modulates immunity and inhibits inflammation, which could improve the long-term effects of cancer treatments [36,37]. ST36 was also found to lower the levels of interleukin-17 (IL-17) and tumor necrosis factor (TNF)-alpha in rat serum [38]. Another recent study found that acupuncture at ST36 dramatically reduced the heart-rate and oxygen-consumption statistics, which indicate fatigue reduction [39]. Acupoint ST36 may also upregulate the level of skeletal-muscle adenosine triphosphate (ATP) synthase and enhance the integration of mitochondrial ATP when the body is afflicted by tiredness or excessive weariness [40]. Therefore, ST36 has been widely used as the primary acupoint in the management of CRF [41,42]. 

While focusing exclusively on AT itself may make it easier to comprehend the effects of a single type of AT, the nondisclosure of evidence from other related therapies may limit the scope of this overview. In order for this overview to be useful for clinicians and researchers, we applied a broader inclusion criterion in order to ensure that all of the available information on all AT and related therapies, such as auricular AT and moxibustion, was included with the data, and was segregated and analyzed according to the AT type.

CRF should be assessed on a regular basis in clinical settings to aid in the identification of appropriate and effective therapies, treatments, and management [31]. Clinicians in busy outpatient practices are limited in their ability to speak about this symptom because of time restrictions. Furthermore, clinicians may be hesitant to mention the potential CRF because of a lack of knowledge in this area and concerns about the treatment alternatives. On the other hand, cancer survivors are sometimes hesitant to disclose fatigue for fear that it could mean a cancer relapse, or that they could be labeled a whiny patient. Some patients also believe that fatigue is a natural side effect of their cancer and treatment. However, because this symptom is becoming more common, and because it can have a significant impact on a patient’s daily life, healthcare practitioners should be encouraged to inquire about CRF, and to pay attention to its management.

Acupuncture can be regarded as a relatively safe therapy, to some extent, when performed properly and by educated acupuncturists. One study’s preliminary findings offer the foundation for the proposition that acupuncture can be utilized safely in cancer patients within an integrated model of oncology care [43]. Another recent study also found that acupuncture is just as safe in cancer patients as sham acupuncture and active controls [44]. 

The overview has some strengths. Firstly, this study is the first overview of SRs/MAs to assess the evidence for the use of AT for CRF. Secondly, the outcomes of the MAs are shown in structured tables, which can help readers to easily review the interesting outcomes. Thirdly, we started this overview with a predesigned protocol, which helped reduce the risk of bias. The overview also has several limitations. First, some of the overlap of the primary articles within the SRs that are included are expected to have duplicates; however, we have not systematically investigated them. This can lead to inaccuracies in the data reporting, such as the numbers of participants and primary studies, and it can contribute to the “double counting” of the reported data. Second, we have not retrieved or analyzed data from any primary studies; we have only relied on information provided by the authors of the SRs. Third, the majority of the SRs/MAs that are included were of poor quality, which lowered the confidence in the evidence, and most of the main study was based primarily on Chinese studies. Finally, this overview did not perform any quantitative analysis, which could result in skewed conclusions.

## 5. Conclusions

The evidence suggests that, despite the advantages of AT in terms of the improvement in and the safety of the treatment of CRF, the methodological quality of most of these studies is low, which limits our ability to draw definitive meanings. Further research is needed to establish firm evidence and further recommendation.

## Figures and Tables

**Table 1:** Characteristics of included systematic reviews/meta-analyses of acupuncture for cancer fatigue.

First Author (Year) [Ref] Country	Search Date/ No. of Searched Databases	No. of Included RCTs (Sample Size)	Intervention	Comparator	Overall Risk of Bias	Overall Confidence Rating	Conclusion (Quote from the Original Paper)	Adverse Events
Tan (2021) [22] Australia	February 2021/13 DBs	15 (1468)	AT/APT	SAT/UC	Moderate to high	Moderate	“…identified a promising role of AT in improving CRF…”	Yes
Jang (2020) [23] Australia	May 2019/4DBs	9 (809)	AT	SAT/UC	Moderate to high	Low	“…suggests that AT has therapeutic potential in management of CRF…”	No
Yuan (2020) [24] China	March 2020/8DBs	11 (832)	AT	APT/SAT/UC	High	Low	“…can effectively…, especially for those who have finished anti-tumor therapy and rarely have adverse effects.”	No
Zhao (2020) [19] China	June 2017/18DBs	5 (547)	AT/Moxa	SAT/UC	Moderate to high	Critically Low	“…to be effective and safe in the treatment of CRF.”	Yes
Zhang (2018) [18] China	November 2016/7DBs	10 (1327)	AT	SAT/UC	Moderate to high	Moderate	“…management and should be recommended as a beneficial alternative therapy…”	Yes
Ling (2014) [25] China	April 2014/16DBs	11 (731)	AT/APT	SAT/UC	n.r.	Critically Low	“…be effective in relieving CRF, with the former producing a greater improvement.”	No
He (2013) [17] China	December 2012/8DBs	7 (804)	AT/APT/Moxa	SAT	Moderate to high	Critically Low	“…appeared to be efficacious auxiliary therapeutic methods…”	Yes
Posadzki (2013) [26] Korea	November 2012/14DBs	7 (548)	AT	SAT/UC	Moderate to high	Critically Low	“…it remained unclear whether the observed outcome was due to specific effects of AT…”	No
Zeng (2014) [27] China	May 2013/5DBs	7 (689)	AT	SAT/UC/Self-AT/ No treatment/Waiting list	Moderate to high	Moderate	“…no statistically significant.”	Yes
Han (2020) [28] China	December 2018/8DBs	6 (394)	APT	SAPT/UC	High	Critically Low	“…may be a safe therapy to relieve CRF and enhance the QoL …”	Yes
Huang (2021) [29] China	July 2020/9BDs	18 (1312)	Moxa	UC	Moderate to high	Critically Low	“…can effectively improve the CRF of patients, improve the QoL …”	Yes
Han (2021) [30] China	May 2020/8BDs	13 (899)	Moxa	UC	Moderate to high	Low	“… can effectively reduce cancer-related fatigue, improve QoL …”	Yes
Hu (2021) [31] China	April 2018/8BDs	28 (2249)	Moxa	UC	Moderate to high	Critically Low	“… safe and effective in treating…”	Yes
Yu (2020) [32] China	April 2018/6BDs	18 (1409)	Moxa	UC	Moderate to high	Low	“…can alleviate the symptoms of CRF and improve the QoL of cancer patients to a certain extent.”	No
Lee (2014) [33] Korea	April 2013/18BDs	4 (374)	Moxa	UC	Moderate to high	Critically Low	“…difficult to draw the conclusion that moxibustion is an effective and safe treatment…”	Yes

APT: auricular point therapy; AT: acupuncture; CRF: cancer-related fatigue; DB: databases; Moxa: moxibustion; RoB: risk of bias; OoL: quality-of-life; SC: standard care; SAPT: sham auricular point therapy; SAT: sham acupuncture; UC: usual care.

**Table 2:** Types of acupoints used for CRF *.

Acupuncture-and-Moxibustion-Related Therapies	No. of Primary Studies (Total/Reported Studies)	Acupuncture Points (No. of Primary Study)
Acupuncture	28/24	ST36 (19), SP6 (15), CV6 (11), CV4 (8)
Acupressure	5/4	LI4(4), SP6 (4), ST36 (4), DU20 (3), CV6 (3), HT7 (3), LR3 (3), KI3 (3)
Auricular acupuncture	7	TF4 (6), AH6a (6), CO12 (4), CO13 (4), CO4 (3)
Moxibustion	46/40	ST36 (26), CV4 (23), CV6 (21), CV12 (13), CV8 (12)
Total	79/68	ST36 (49), CV6 (35), CV4 (32), SP6 (21), CV12 (15), CV8 (12), LI4 (11)

* The most frequently used (more than 30% of the included studies in each intervention) acupuncture points.

**Table 3:** Quality of evidence in the included SRs assessed by the GRADE approach.

First Author (Year)	Outcomes	Number of RCTs (Number of Participants)	Relative Absolute (95% CI)	*p*-Value	Quality of Evidence
Tan (2021) [22]	Fatigue (short-term) (AS vs. UC)	8 (426)	SMD −0.95 [−1.72, −0.18]	0.02	Low
	Fatigue (medium-term) (AS vs. UC)	2 (44)	SMD −0.96 [−1.60, −0.33]	0.003	Very low
	Fatigue (medium-term) (AS vs. SAS)	2 (133)	SMD −0.29 [−0.65, −0.07]	0.11	Very low
	Fatigue (AT vs. UC)	7 (361)	SMD −1.25 [−2.05, −0.45]	0.0002	Low
	Fatigue (AT vs. SAT)	2 (123)	SMD −0.29 [−0.65, −0.07]	0.11	Very low
	Fatigue (acupressure vs. sham acupressure)	2 (100)	SMD −0.26 [−0.66, −0.14]	0.20	Very low
Jang (2020) [23]	Fatigue (BFI) (AT vs. SAT)	6 (189)	SMD −0.93 [−1.65, −0.20]	<0.00001	Low
	Fatigue (BFI) (AT vs. UC)	3(78)	SMD −2.12 [−3.21, −1.04]	<0.00001	Low
Yuan (2020) [24]	Fatigue	11 (832)	SMD −1.06 [−1.73, −0.40]	0.002	Moderate
	Fatigue (F/U)	3 (129)	SMD −0.85 [−2.86, 1.16]	0.41	Very low
	QoL	4 (187)	SMD 0.26 [−0.03, 0.55]	0.08	Very low
Zhao (2020) [19]	Fatigue	5 (547)	SMD 0.48 [0.30, 0.66]	<0.00001	Moderate
Zhang (2018) [18]	Fatigue	10 (1327)	SMD −1.26 [−1.80, −0.71]	<0.00001	Low
He (2013) [17]	Fatigue	2 (198)	OR 0.16 [0.07, 0.37]	<0.00001	Very low
Zeng (2013) [27]	Fatigue (AT vs. SAT)	3 (121)	SMD −0.82 [−1.90, 0.26]	0.14	Very low
	Fatigue (AT vs. UC)	2 (314)	SMD −2.12 [−3.21, −1.03]	0.001	Very low
	Fatigue (AT vs. no treatment)	2 (150)	SMD −1.46 [−3.56, 0.63]	0.17	Very low
	Fatigue (AT vs. other treatment)	2 (163)	SMD −1.12 [−3.03, 0.78]	0.17	Very low
	QoL	3 (121)	SMD 0.99 [−0.70, 2.68]	<0.00001	Very low
	Functional well-being	3 (121)	SMD 1.38 [−1.02, 3.79]	<0.00001	Very low
Han (2020) [28]	Fatigue	5 (170)	RR 1.76 [1.42, 2.17]	<0.00001	Low
	QoL	3 (215)	MD 7.34 [5.11, 9.57]	<0.00001	Low
Huang (2021) [29]	Fatigue	15 (1040)	SMD −1.30 [−1.44, −1.16]	<0.00001	Low
	QoL	9 (572)	SMD 1.39 [0.87, 1.90]	<0.00001	Low
Han (2021) [30]	Fatigue	13 (892)	SMD −1.58 [−2.05, −1.11]	<0.00001	Low
	QoL	5 (283)	MD 11.50 [7.94, 15.06]	<0.00001	Very low
	TCM syndrome	4 (298)	MD −1.21 [−1.58, −0.84]	<0.00001	Very low
Hu (2021) [31]	Fatigue (response rate)	6 (538)	OR 5.21 [2.66, 10.19]	<0.00001	Low
	Fatigue	15 (1147)	MD 1.91 [1.29, 2.52]	<0.00001	Low
Yu (2020) [32]	Fatigue (PFS)	8 (668)	MD −1.29 [−1.88, −0.70]	<0.001	Moderate
	Fatigue (BFI)	5 (438)	MD −0.93 [−2.72, −0.86]	<0.00001	Very low
	Fatigue (KPS)	10 (714)	MD 7.08 [3.31, 10.85]	<0.00001	Low
	QoL	5 (365)	MD 9.88 [5.03, 14.73]	<0.00001	Very low
Lee (2014) [33]	Fatigue (response rate)	4 (340)	RR 1.73 [1.29, 2.32]	0.0003	Very low

AS: acupoint stimulation; AT: acupuncture; BFI: brief fatigue inventory; KPS: Karnofsky performance scale; MD: mean difference; F/U: follow-up; OR: odds ratio; PFS: Piper fatigue scale; QoL: quality of life; RR: risk ratio; SAS: sham acupoint stimulation; SAT: sham acupuncture; SMD: standardized mean difference; WMD: weighted mean difference; UC: usual care.

## Supplementary Materials

The following supporting information can be downloaded at: https://www.mdpi.com/article/10.3390/cancers14102347/s1, Table S1: The list of excluded studies with reasons; Table S2: Assessment of all included systematic reviews using AMSTAR 2.
