# Supplementary material for: Acupuncture and Moxibustion for Cancer-Related Fatigue: An Overview of Systematic Reviews and Meta-Analysis
Source: Cancers (Basel). 2022 May 10;14(10):2347. doi: 10.3390/cancers14102347 (PMC9139178; doi:10.3390/cancers14102347)
Supplement: Supplementary file 1 [file cancers-14-02347-s001.zip › cancers-1675102-supplementary.pdf]

Table S1. The list of excluded studies with reasons.

Citation	Reason for exclusion
Finnegan-John J, Molassiotis A, Richardson A, Ream E. A systematic review of complementary and alternative medicine interventions for the management of cancer-related fatigue. *Integr Cancer Ther* 2013;12:276-90.	Included acupuncture and other treatments
Grant SJ, Smith CA, de Silva N, Su C. Defining the quality of acupuncture: the case of acupuncture for cancer-related fatigue. *Integr Cancer Ther* 2015;14:258-70.	Not a systematic review
Han QQ, Fu Y, Le JM, et al. The therapeutic effects of acupuncture and electroacupuncture on cancer-related symptoms and side-effects. *J Cancer* 2021;12:7003-9.	Not a systematic review
David A, Hausner D, Frenkel M. Cancer-related fatigue-Is there a role for complementary and integrative medicine? *Curr Oncol Rep* 2021;23:145.	Not a systematic review/ Included acupuncture and other treatments
Huang Z, Zhang Q, Fan Y, et al. Effect of traditional chinese medicine injection on cancer-related fatigue: a meta-analysis based on existing evidence. *Evid Based Complement Alternat Med* 2020;2020:2456873.	Included acupuncture and other treatments
Chau HY. The study of meta-analysis of acuncture for cancer-related fatiue. *Guangzhou University of Chinese Medicine* 2017;Doctor degree.	Dissertation/ Duplicate publication
Hu Y. Application research of ChuZhen and moxibustion therapy based on the theory of FuZheng in patients with cancer-related fatigue. *Chengdu University of Chinese Medicine* 2020;Master´s degree.	Dissertation/ Duplicate publication

Table S2. Assessment of all included systematic reviews using AMSTAR 2.

**First author (year)**	**1**	**2**	**3**	**4**	**5**	**6**	**7**	**8**	**9**	**10**	**11**	**12**	**13**	**14**	**15**	**16**	**Rating overall confidence***
Tan (2021) [18]	Y	Y	N	Y	Y	Y	N	Y	Y	N	Y	Y	Y	Y	N	Y	Moderate
Jang (2020) [19]	Y	N	N	PY	Y	Y	N	Y	Y	N	Y	Y	Y	N	N	Y	Low
Yuan (2020) [20]	Y	N	N	Y	Y	Y	N	Y	Y	N	Y	Y	Y	Y	N	N	Low
Zhao (2020) [16]	Y	N	N	Y	Y	Y	N	Y	Y	N	Y	Y	Y	N	N	Y	Critically Low
Zhang (2018) [15]	Y	N	N	Y	Y	Y	N	Y	Y	N	Y	Y	Y	Y	N	Y	Moderate
Ling (2014) [21]	Y	N	N	Y	N	N	N	Y	Y	N	NMA	NMA	Y	NMA	NMA	Y	Critically Low
He (2013) [14]	Y	N	N	Y	N	N	N	N	Y	N	Y	N	Y	N	N	Y	Critically Low
Posadzki (2013) [22]	Y	N	N	Y	Y	Y	N	Y	Y	N	NMA	NMA	Y	NMA	NMA	Y	Critically Low
Zeng (2014) [23]	Y	N	N	PY	Y	Y	N	Y	Y	N	Y	Y	Y	Y	N	Y	Moderate
Han (2020) [24]	Y	N	N	Y	Y	N	N	Y	Y	N	Y	N	Y	N	N	Y	Critically Low
Huang (2021) [25]	Y	N	N	Y	Y	N	N	Y	Y	N	Y	N	Y	Y	N	N	Critically Low
Han (2021) [26]	Y	N	N	Y	N	Y	N	Y	Y	N	Y	Y	Y	N	Y	N	Low
Hu (2021) [27]	Y	N	N	Y	N	Y	N	Y	Y	N	Y	N	Y	N	No	N	Critically Low
Yu (2020) [28]	Y	N	N	Y	Y	Y	N	Y	Y	N	Y	Y	Y	N	Y	N	Low
Lee (2014) [29]	Y	N	N	Y	Y	N	N	Y	Y	N	Y	N	Y	N	N	Y	Critically Low
No. of Y (%)	15 **(100)**	1 **(6.6)**	0 **(0)**	13 **(86.6)**	11 **(73.3)**	10 **(66.6)**	0 **(0)**	14 **(93.3)**	15 **(100)**	0 **(0)**	13 **(86.6)**	8 **(53.3)**	15 **(100)**	5 **(33.3)**	2 **(13.3)**	10 **(66.6)**	

Y: Yes; PY: Partial Yes; N: No; NMA: No meta-analysis conducted.

Important items 2, 4, 7, 9, 11, Questions 13 and 15 are of very low quality if two or more of these items are omitted, low quality if one important item is omitted, moderate quality if two or more of the non-critical items other than the above important items are omitted, no missing items or non-important If one item is omitted, it is rated as excellent quality.

Domains:

1. Did the research questions and inclusion criteria for the review include the components of PICO?
2. Did the report of the review contain an explicit statement that the review methods were established prior to the conduct of the review and did the report justify any significant deviations from the protocol?
3. Did the review authors explain their selection of the study designs for inclusion in the review?
4. Did the review authors use a comprehensive literature search strategy?
5. Did the review authors perform study selection in duplicate?
6. Did the review authors perform data extraction in duplicate?
7. Did the review authors provide a list of excluded studies and justify the exclusions?
8. Did the review authors describe the included studies in adequate detail?
9. Did the review authors use a satisfactory technique for assessing the risk of bias (RoB) in individual studies that were included in the review?
10. Did the review authors report on the sources of funding for the studies included in the review?
11. If meta-analysis was performed did the review authors use appropriate methods for statistical combination of results?
12. If meta-analysis was performed, did the review authors assess the potential impact of RoB in individual studies on the results of the meta-analysis or other evidence synthesis?
13. Did the review authors account for RoB in individual studies when interpreting/discussing the results of the review?
14. Did the review authors provide a satisfactory explanation for, and discussion of, any heterogeneity observed in the results of the review?
15. If they performed quantitative synthesis did the review authors carry out an adequate investigation of publication bias (small study bias) and discuss its likely impact on the results of the review?
16. Did the review authors report any potential sources of conflict of interest, including any funding they received for conducting the review?
